# HuMiTar: A sequence-based method for prediction of human microRNA targets

**DOI:** 10.1186/1748-7188-3-16

**Published:** 2008-12-22

**Authors:** Jishou Ruan, Hanzhe Chen, Lukasz Kurgan, Ke Chen, Chunsheng Kang, Peiyu Pu

**Affiliations:** 1 Chern Institute for Mathematics, College of Mathematics and LPMC, Nankai University, Tianjin, PR China; 2 Department of Electrical and Computer Engineering, University of Alberta, Canada; 3 Neuro-oncology laboratory, General Hospital of the Tianjin Medical University, Tianjin, PR China

## Abstract

**Background:**

MicroRNAs (miRs) are small noncoding RNAs that bind to complementary/partially complementary sites in the 3' untranslated regions of target genes to regulate protein production of the target transcript and to induce mRNA degradation or mRNA cleavage. The ability to perform accurate, high-throughput identification of physiologically active miR targets would enable functional characterization of individual miRs. Current target prediction methods include traditional approaches that are based on specific base-pairing rules in the miR's seed region and implementation of cross-species conservation of the target site, and machine learning (ML) methods that explore patterns that contrast true and false miR-mRNA duplexes. However, in the case of the traditional methods research shows that some seed region matches that are conserved are false positives and that some of the experimentally validated target sites are not conserved.

**Results:**

We present HuMiTar, a computational method for identifying common targets of miRs, which is based on a scoring function that considers base-pairing for both seed and non-seed positions for human miR-mRNA duplexes. Our design shows that certain non-seed miR nucleotides, such as 14, 18, 13, 11, and 17, are characterized by a strong bias towards formation of Watson-Crick pairing. We contrasted HuMiTar with several representative competing methods on two sets of human miR targets and a set of ten glioblastoma oncogenes. Comparison with the two best performing traditional methods, PicTar and TargetScanS, and a representative ML method that considers the non-seed positions, NBmiRTar, shows that HuMiTar predictions include majority of the predictions of the other three methods. At the same time, the proposed method is also capable of finding more true positive targets as a trade-off for an increased number of predictions. Genome-wide predictions show that the proposed method is characterized by 1.99 signal-to-noise ratio and linear, with respect to the length of the mRNA sequence, computational complexity. The ROC analysis shows that HuMiTar obtains results comparable with PicTar, which are characterized by high true positive rates that are coupled with moderate values of false positive rates.

**Conclusion:**

The proposed HuMiTar method constitutes a step towards providing an efficient model for studying translational gene regulation by miRs.

## Background

MicroRNAs (miRs) are endogenously expressed non-coding RNAs, which downregulate expression of their target mRNAs by inhibiting translational initiation or by inducing degradation of mRNA [1]. They are associated with numerous gene families in multi-cellular species and their regulatory functions in various biological processes are widespread [2-14]. The ability to perform accurate, high-throughput identification of physiologically active miR targets is one of the enabling factors for functional characterization of individual miRs. This is also true in case on human miRs, for which only a handful have been experimentally linked to specific functions. The methods for the prediction of miR targets can be subdivided into two classes, traditional approaches, which combine several factors such as sequence complementarity, minimization of free energy, and cross-species conservation, and machine learning (ML) methods that exploit statistical patterns that differentiate between true and false miR-mRNA duplexes. The former methods aim at finding target sites for a given miR by scanning 3' untranslated region (UTR) of the mRNA, while the latter methods classify a given duplex as true or false.

Current traditional sequence-based target predictors are based on the presence of a conserved 'seed region' (nucleotides 2–7) of exact Watson-Crick complementary base-pairing between the 3' UTR of the mRNA and the 5' end of the miR [15,16]. They are based on two principles: (1) identification of potential miR binding sites according to specific base-pairing rules in the seed region, and (2) implementation of cross-species conservation [17]. Recent survey by Sethupathy and colleagues [18] compared five widely used traditional tools for mammalian target prediction which include DIANA-microT [7], miRanda [19], TargetScan [3], TargetScanS [11], and PicTar [10]. They observed that the earlier methods, i.e., TargetScan and DIANA-microT, achieve a relatively low sensitivity and predict a small number of targets. The miRanda was shown to provide a substantially better sensitivity as a trade-off for large increase in the total number of predictions. The two more recent programs, TargetScanS and PicTar, have almost identical sensitivity when compared with miRanda but they predict several thousand fewer miR-mRNA interactions. Another survey that investigated several traditional predictors including PicTar, TargetScanS, miRanda, and RNAhybrid [8], concludes that miRanda and RNAhybrid obtain lower accuracy and sensitivity when compared with TargetScanS and PicTar [17]. These conclusions were also confirmed in a recent study by Huang and colleagues [16]. They show that the highest quality predictions are obtained by TargetScanS, closely followed by PicTar, while miRanda and DIANA-microT were ranked lower. Most recently, Kuhn and colleagues suggest use of PictTar, TargetScanS, and PicTar to perform computational prediction of miR targets [20]. Based on the above, our experimental section includes three representative miR target prediction methods, TargetScanS, PicTar, and Diana-MicroT. The first two were selected based on their favorable performance, while predictions of Diana-MicroT were used as a point of reference, i.e., representative early generation program characterized by a relatively low sensitivity.

Recent research resulted in development of several ML methods. These methods usually filter predictions provided by the traditional predictors. Their main drawback is that they filter targets by using a predefined and relatively small number of false targets, i.e., they do not scan the mRNA sequence but instead they simulate that by using a small set of negatives (false targets). For instance, a method by Yan and colleagues filters miRanda's predictions based on 48 positive and 16 negative sites [21]. A more recent, NBmiRTar method, which also filters predictions of miRanda, applies 225 true miR targets, 38 confirmed negative sites, and up to 5000 of artificially generated negative sites [22]. The most recent method is based on binding matrix technique, in which the information concerning both the miRNA sequence and a set of experimentally validated targets is used to perform predictions [23]. The main drawback of this approach is the necessity of providing a set of validated targets which is not required in case of the proposed and the abovementioned sequence-based prediction methods. At the same time, we note that the ML methods establish the prediction model based on information concerning both the seed and the non-seed positions, which is also exploited in our research. To this end, we include NBmiRTar method in our experimental section.

We aim at developing a novel, traditional prediction method, named HuMiTar, which addresses some of the drawbacks of the existing seed-based methods. Although the existing methods strongly emphasize the seed-region complementarity and the cross-species conservation, as many as 40% of seed region matches that are conserved between human and chicken are false positives [11], and imperfect pairing is shown to occur in the seed region [24]. Another recent study indicates that almost 30% of the experimentally validated target sites are not conserved, motivating the development of alternative computational methods [18]. Although relaxation of the conservation results in higher sensitivity, it also leads to higher false positive rates, which in turn results in necessity of performing extensive laboratory verification on the predicted interactions [16]. A recently proposed solution to increase quality of traditional predictors is based on filtering predictions of sequence-based methods using profiling of miR and mRNA expressions [16]. We propose an alternative approach, in which instead of filtering results of existing sequence-based methods (as done by the ML methods), we develop a novel sequence-based design that aims at improving true positive rates. We collected statistical information using a design dataset of 66 human miR-mRNA duplexes that were published in TarBase [25] before 2006, see Table 1 [see Additional file 1]. HuMiTar incorporates two main components which are designed based on a quantitative analysis of these duplexes: (1) a novel composite scoring function that quantifies strength of miR-mRNA binding and which incorporates information about base-pairing for both seed and non-seed positions; and (2) a 2D-coding method that finds potential targets in 3'UTR which are next scored and filtered via the scoring function. Improved prediction quality of the proposed method is a result of a careful design and optimization that is focused on human targets. The motivation to choose human targets comes from two facts: (1) target prediction for plants is easier than for animals [26,27]; and (2) identification of miR targets is critical to advancing understanding of human diseases, such as cancer, arising from misregulation of gene expression caused by miRs [28]. At the same time, to date, a relatively small number of target genes in various tumors was experimentally identified for some miRs [29].

**Table 1:** Datasets.

Dataset name	Dataset details	Dataset goal
*Design set*	66 human miR-mRNA duplexes published in TarBase before 2006, see Table 1 [see Additional file 1]; this set includes 29 miRs and 36 genes.	Design of the proposed prediction method. Evaluation and comparison of sensitivity – # predictions trade-off and overlap between the predictions of different methods.

*Independent set*	39 human miRs that were published in TarBase between January 2006 and June 2007; this set includes 20 miRs and 26 genes.	Evaluation and comparison of sensitivity – # predictions trade-off and overlap between the predictions of different methods.

*Interactions set*	190 miR-mRNA interactions pairs experimentally tested in *Drophilia*. The dataset was taken from [30].	Evaluation and comparison of the specificity/sensitivity trade-off. The ROC curves and AUC values were compared with results of five competing methods reported in [30].

*GO *(glioblastoma oncogenes) *set*	Ten glioblastoma oncogenes. The choice is motivated by our expertise in profiling glioblastoma. Although our goal was to compare predictions on 17 glioblastoma oncogenes, only 10 of them could be found in PicTar database. The oncogenes and the associated 328 miRs are given in Tables 2 and 3 [see Additional file 1], respectively.	Comparison of the sensitivity, number of predicted targets and overlap between the predictions of different methods.

## Results and discussion

### Datasets and experimental setup

Dataset used to validate and compare the proposed prediction method are summarized in Table 1. The following empirical tests were performed:

*1. Comparison of sensitivity – number of predicted targets trade-off*. HuMiTar, PicTar, DIANA-MicroT, TargetScanS, and NBmiRTar were compared on the design set and the independent set.

*2. Comparison of the overlap of predictions*. HuMiTar is compared with the best-performing traditional method PicTar and TargetScanS and ML method NBmiRTar on the GO set. We also include Western blots which are used to verify correctness of some of the HuMiTar predictions.

*3. Evaluation and comparison of sensitivity/specificity trade-off *based on ROC (receiver operating characteristic) analysis. The predictions of HuMiTar on the interactions set were compared with results of five competing predictions methods reported in [30].

*4. Predictions on p53*. HuMiTar predicted a set of miRs that target p53, some of which were independently verified in [31].

*5. Genome-wide target prediction*. HuMiTar was applied to perform genome wide predictions for 16 different species.

We also estimate the signal-to-noise ratio based on predictions for human 3' UTRs and using the procedure introduced to validate PicTar [10]. This ratio and the analysis presented in test 1 are performed to estimate specificity of the proposed method; similar evaluation for the traditional methods was done in [3,10,11,17,18]. Finally, we also estimate the computational complexity of the proposed method.

### Comparison of sensitivity – number of predicted targets trade-off

**Figure 1:**
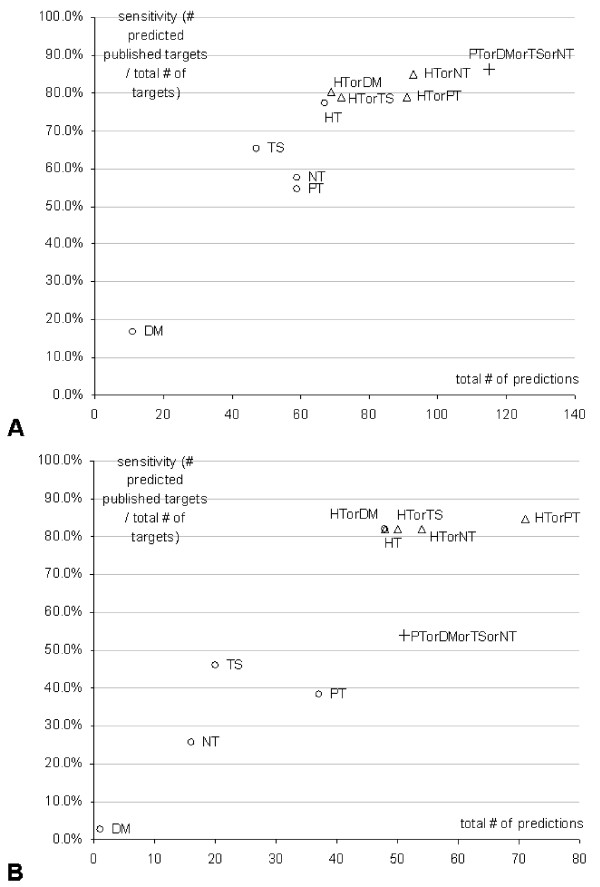
**Summary of prediction results of HuMiTar (HT), PicTar (PT), DIANA-MicroT (DM), TargetScanS (TS), and NBmiRTar (NT). Panel A gives results for the design set of 66 miR-mRNA duplexes. Panel B gives results for the independent set of 39 miR-mRNA duplexes**. The hollow circles show results of individual methods, hollow triangles show results of union between HuMiTar and one competing method, and cross corresponds to union of the four competing methods.

Detailed results for the five prediction methods (HuMiTar, PicTar, DIANA-MicroT, TargetScanS, and NBmiRTar) and each of the miRs in the design and independent sets are listed in Tables 4 and 5 [see Additional file 1], respectively. The predictions are summarized in Figure 1. Following the analysis performed in [18], the Figure shows sensitivity (number of predicted published targets divided by total number of published targets) against the total number of predictions. We show results for each of the five methods, and also when combining (using union) predictions of HuMiTar with each of the competing method, as well as for the union of the four competing methods. This allows not only to analyze sensitivity-specificity trade-off, as defined in [18], but also to investigate complementarity between predictions of different methods. We note that increased sensitivity comes at a price of the increased number of predictions. We also note that TargteScanS and PicTar have comparable sensitivity, while the sensitivity of DIANA-MicroT is much lower, which agrees with the conclusions from [18]. We observe that: (1) HuMiTar provides the highest sensitivity among the five predictors as a trade-off for a moderate increase of the number of predicted targets, i.e., 67 vs. 59/47 targets were predicted by PicTar/TargetScanS for the design set and 48 vs. 37/20 were predicted by PicTar/TargetScanS for the independent set; (2) DIANA-MicroT is shown to provide the lowest sensitivity and low number of predictions; (3) TargetScanS provides the second best sensitivity with a relatively low number of predicted targets; (4) NBmiRTar obtains results comparable to PicTar on the design set and a relatively low sensitivity on the independent set; (5) addition of predictions of competing methods to predictions of HuMiTar results in small or no improvement in sensitivity while it increases the total number of predictions; (6) union of the competing four predictors on the independent set shows relatively low sensitivity with similar number of predicted targets when compared with HuMiTar. The last two findings indicate that HuMiTar is capable of providing additional true positive predictions as a trade-off for a moderate increase in the number of predicted targets. The largest number of 23 (design set) and 22 (independent set) unpublished targets, i.e., targets found for some of the miRs from the design/independent set that are not published in the TarBase, was found by PicTar. These targets may correspond to biologically meaningful sites or may constitute false positive predictions. The five methods predict relatively low number of unpublished targets, especially in the context of the generated number of true positives and the fact that PicTar was previously shown to provide relatively low false positive rates [10].

Following, we concentrate on the results on the independent set since this set was not used to design the proposed method and thus it allows for an unbiased analysis. A recent study by Nielsen and colleagues reveals several miR targeting determinants [32]. They concern patterns outside of the seed and include presence of adenosine opposite miR base 1 and of adenosine or uridine opposite miR base 9. We applied both of these determinants on the set of 39 duplexes, and found 10 matches, i.e., 10 duplexes satisfy both of the determinants. All of these 10 duplexes were correctly predicted by HuMiTar, while 8 were predicted by TargetScanS, 6 by PicTar, 5 by NBmiRTar, and none by DIANA-MicroT. When considering 13 out of 39 cases for which the adenosine or uridine was opposite miR base 9, all of them were correctly classified by HuMiTar, and 10, 8, 6, and 0 by TargerScanS, PicTar, NBmiRTar, and DIANA-MicroT, respectively. Finally, for 19 duplexes in which the adenosine was opposite miR base 1, 16 of them were found by HuMiTar, 12 by TargerScanS, 10 by PicTar, 7 by NBmiRTar, and none by DIANA-MicroT. This provides an independent validation of the improvements provided by the HuMiTar, which uses scoring function that considers base-pairing outside of the seed, in contrast to the traditional methods that are based on the base-pairing rules only in the seed region.

### Comparison of the overlap of predictions

**Figure 2:**
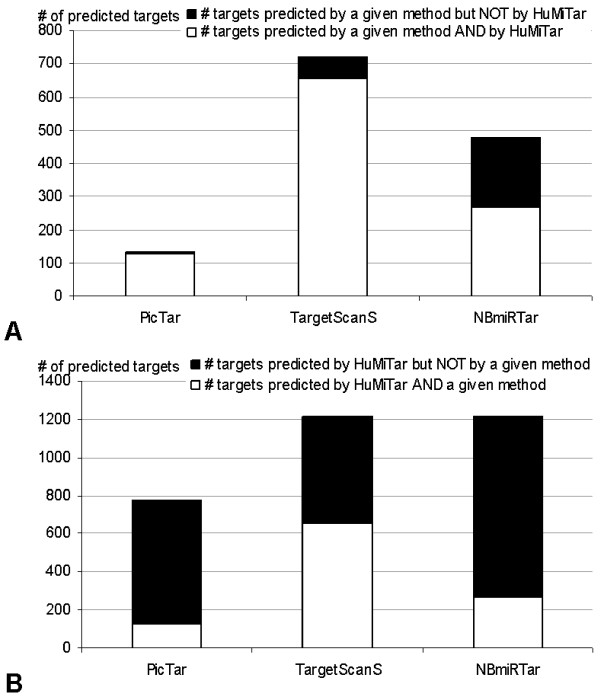
**Comparison of the number of predicted targets and their overlap for predictions on the GO set. Panel A shows the number of targets predicted by the three competing methods including PicTar, TargetScanS, and NBmiRTar. The white area represents targets that overlap with predictions of HuMiTar and the black area shows the remaining targets. Panel B shows the number of targets predicted by HuMiTar. The white area shows overlap with predictions of a competing method indicated at the *x*-axis, and the black area shows predictions specific to HuMiTar**. In the case of PicTar, the predictions are reduced to a set of miRs that are included in the PicTar's database .

328 human miRs were predicted on the GO set with PicTar, TargetScanS, NBmiRTar, and HuMiTar to analyze overlap between predictions of different methods. The results are summarized in Figure 2. The detailed results (including values for individual oncogenes) that compare HuMiTar with PicTar, TargetScanS, and NBmiRTar are provided in Tables 6, 7, and 8 [see Additional file 1], respectively. Since PicTar's database does not include some of the miRs considered in this test, they were excluded from the comparison with this method, which results in a lower total number of PicTar's predictions. Following we compare the predictions of HuMiTar with each of the three competing methods.

HuMiTar predicts 97% of the targets that were predicted by PicTar while only 4 targets were predicted exclusively by PicTar. At the same time, HuMiTar finds numerous extra targets that were not predicted by PicTar. Among them, 646 and 442 extra targets were predicted for miRs that are and that are not included in the PicTar's database, respectively. Although the results show that HiMiTar generates larger number of predictions that cover virtually all predictions made by PicTar, these additional prediction could constitute either true or false positives. Since it would infeasible to verify correctness of the entire set of 1088 additional predictions provided by HuMiTar, we concentrate our efforts on a specific target, Septin7, due to our existing wet-lab expertise. The test considers three sets of miRs:

#### Set 1

5 miRs from the 18 targets that were predicted by both HuMiTar and PicTar, i.e. miR-19a, miR-127, miR-141, miR-182, and miR183. This set of used to investigate whether the common results in fact concern true positives.

#### Set 2

5 miRs from the 23 targets that were predicted only by HuMiTar and which are not included in the PicTar's database, i.e. miR-202, miR-248, miR-412, miR-453, and miR-450. This set concerns miRs that have not been predicted by PicTar, i.e. they were predicted only with the use of HuMiTar.

#### Set 3

11 miRs from the 34 extra targets on Septin7, which were found only by HuMiTar although these miRs are included in PicTar's database, i.e. miR-148, miR-106b, miR-134, miR-106, miR-144, miR-151, miR-384, miR-101, miR-142, miR-129, and miR-126. This set is of particular interest (and thus it is larger), since it concerns targets that were not predicted by PicTar, but which were predicted by HuMiTar.

We run a Western blot according to the following procedure. The human glioblastoma cell line U251 was obtained from China Academia Sinica cell repository in Shanghai, China. All cell lines were grown in Dulbecco's modified Eagle's medium (DMEM) (Gibco, USA) supplemented with 10% fetal bovine serum (Gibco, USA), 2 mM glutamine (Sigma, USA), 100 units of penicillin/ml (Sigma, USA), and 100 μg of streptomycin/ml (Sigma, USA), incubated at 37°C with 5% CO2, and sub-cultured every 2~3 days. The antisense oligonucleotides of the pre-scanned miRNAs were chemically synthesized by GenePharma (Shanghai, China) and were transfected into U251 cells by Oligofectamine (Invitrogen, USA) according to the manufactures' protocol. Parental and transfected cells were washed with ice-cold phosphate-buffered saline (PBS) three times. The cells were then solubilized in 1% Nonidet P-40 lysis buffer (20 mM Tris, pH 8.0, 137 mM NaCl, 1% Nonidet P-40, 10% glycerol, 1 mM CaCl_2_, 1 mM MgCl_2_, 1 mM phenylmethylsulfonyl fluoride, 1 mM sodium fluoride, 1 mM sodium orthovanadate, and a protease inhibitor mixture). Homogenates were clarified by centrifugation at 20,000 ×g for 15 minutes at 4°C and protein concentrations were determined by a bicinchoninic acid protein assay kit (Pierce, USA). Equal amounts of lysates were subjected to SDS-PAGE on 8% SDS-acrylamide gel. Separate proteins were transferred to PVDF membranes (Millipore, USA) and incubated with primary antibody against Septin-7 (Santa Cruz, USA), followed by incubation with HRP-conjugated secondary antibody (Zymed, USA). The specific protein was detected by using a SuperSignal protein detection kit (Pierce, USA). The membrane was stripped and re-probed with antibody against β-actin (Santa Cruz, USA).

**Figure 3:**
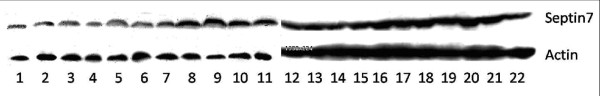
**Western blots for selected 21 miRs and Septin7**. The Septin7 expression levels were measured (left to right) for (1) control sample, (2) miR-127, (3) miR-182, (4) miR-412, (5) miR-19a, (6) miR-453, (7) miR-448, (8) miR-450, (9) miR-183, (10) miR-141, (11) miR-202, (12) miR-148, (13) miR-106b, (14) miR-134, (15) miR-106, (16) miR-144, (17) miR-151, (18) miR-384, (19) miR-101, (20) miR-142, (21) miR-129 and (22) miR-126. The position 1 is the control sample; positions 2 to 11 inclusive concern 10 miRs for which predictions were obtained either by both PicTar and HiMiTar (positions 2, 3, 5, 9, and 10) or only by HuMiTar while these MiR were not included in the PicTar's database (positions 4, 6, 7, 8, and 11); positions 12 to 22 concern 11 miRs which were predicted by HuMiTar and which were included in the PicTar's database. We note that our analysis lacks results on the mutant targets that would strengthen the claim that the activation results of up-regulation of the predicted miRs. Due to limited resources and since the goal of this work is to present a new in-silico prediction method rather than to investigate whether miRs can up-regulate translation, we note that our conclusions concerning the up-regulation should not be considered as the primary outcome of this work.

The Western blot given in Figure 3 shows that:

- In Set 1, up-regulation is shown for miR-19a (position 5), miR-183 (position 9), and miR-141 (position 10); we also observe that miR-182 (position 3) is likely to be a true positive. The experiment shows that miR-127 does not affect the expression levels of Septin7, which may suggest that this is a false positive prediction. To summarize, 4(3) out of 5 of the predictions in Set 1 are shown to be true positives.

- In Set 2, miR-448 (position 7), miR-450 (position 8), and miR-202 (position 11) show up-regulation; miR-453 (position 6) is a borderline case, although the Western blot suggests that it could be classified as a true positive. Finally, miR-412 has no impact on the Septin7 expression, and thus it should be considered as a false positive. As a result, 4(3) out of 5 predictions in this set are true positives.

- In Set 3, the Western blots indicate that all 11 miRs (positions 12 to 22 in Figure 3) target Septin7 and thus they constitute true positive predictions.

Overall, the experiment indicates that for the considered set of miRs, HuMiTar obtains about 80% sensitivity when predicting targets on Septin7. The reported up-regulation is consistent with recent research that also indicates that miRs can up-regulate the translation [33,34]. Although the above results cannot be generalized to other targets, they indicate that predictions generated by the proposed method are characterized by favorable sensitivity when compared with PicTar.

Among the TargetScanS predictions, 91% were also predicted by HuMiTar and the remaining 9%, i.e., 68 targets, were not included in the output of HuMiTar. At the same time, the proposed method provides 562 additional predictions. Similarly as in case of comparison with PicTar, we probe the sensitivity of both prediction methods based on targets predicted for Septin7. Analysis of 39 miRs that were predicted exclusively by HuMiTar shows that 11 of them (miR-101, miR-126, miR-129, miR-134, miR-144, miR-151, miR-202, miR-384, miR-412, miR-450, miR-453) are included in the Western blot on Figure 3. Among them, nine are true positives, miR-453 is a borderline case, and miR-412 is a false positive. Although our limited resources prohibit more extensive experimental analysis, the above analysis suggests that additional predictions provided by HuMiTar include true positives.

Finally, although the overlap between the predictions of HuMiTar and NBmiRTar is the smallest among the three competing methods, it still constitutes over a half (56%) of the NBmiRTar's predictions. The HuMiTar provides 949 predictions which are not included in the output of NBmiRTar, while 213 predictions are exclusive to NBmiRTar.

Overall, the test on the GO set shows that predictions of HuMiTar overlap with the predictions of the competing methods. In particular, we note that the HuMiTar's outputs cover almost all predictions of PicTar and majority of predictions of the other two methods. At the same time, our predictions also include novel targets that could correspond to biologically meaningful sites.

### Evaluation and comparison of sensitivity/specificity trade-off

**Figure 4:**
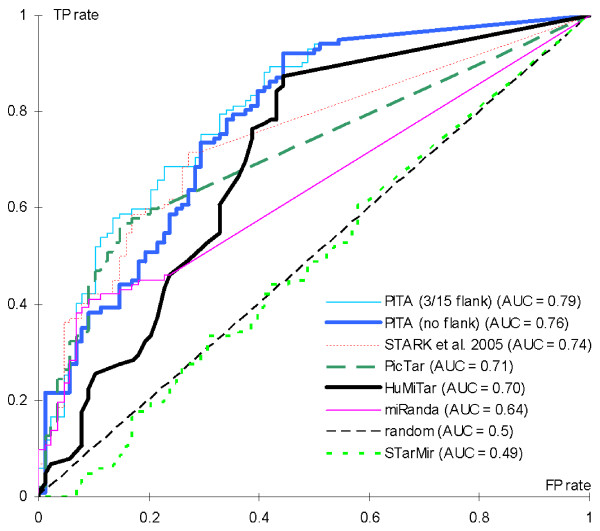
**ROC curves and the corresponding AUC scores that quantify sensitivity and specificity of different miR target predictors on the interactions set**. The results include five existing prediction methods, PicTar [10], miRanda [19], method by Stark and colleagues [35], STarMir [36], and PITA [30]. The latter method includes two versions, one requiring unpairing of only target-site nucleotides (PITA no flank) and another that also requires unpairing of 3 and 15 flanking nucleotides upstream and downstream of the target site (PITA 3/15 flank), respectively. For each prediction method, the targets were sorted by score and the FP rates (*x *axis) and TP rates (*y *axis) were plotted for each possible score prediction threshold. The area under the curve (AUC) for each method is shown in the figure legend. The AUC is computed by extending each plot to the upper right corner as in [30]. The results obtained by a random sorting of the targets are shown using a thin dashed line. The ROC curves and AUC values of PITA, STARK, PicTar, miRanda, and STarMir were taken from [30].

We use the interactions set from [30] to investigate and compare the trade-off between sensitivity and specificity of HuMiTar and several existing methods. This test differs from the other tests shown in this contribution as it simplifies this prediction problem to finding whether a given miR interacts with a given mRNA, i.e., the prediction of the exact location of the target site is ignored. The miR-mRNA pairs from the interactions set are reported in a binary format, as being either functional or non-functional, and we use the area under the ROC curve (AUC) measure to evaluate the sensitivity and specificity of our prediction method. We predict all potential sites in the 3'UTR regions for a given miR and we use the maximal score computed by the scoring function to decide whether the interaction occurs. The ROC curve, see Figure 4, shows the trade-off between the true positive (TP) rate (number of correct predictions of the functional miR-mRNA pairs divided by the total number of functional pairs) and the false positive (FP) rate (number of miR-mRNA pairs that were incorrectly predicted as functional divided by the total number of non-functional pairs) obtained by thresholding the scores. HuMiTar is compared against five other predictions methods, PicTar, miRanda, predictor proposed by Stark and colleagues in [35], STarMir [36], and PITA [30], which were reported in [30]. The proposed method achieves AUC equal 0.70, which is better than the results of STarMir and MiRanda and comparable to results of PicTar and the method by Stark et al. We emphasize that some of these methods use information concerning conservation of the sites in related species, while the only inputs for HuMiTar are the mRNA and MiR sequences. Although HuMiTar is outperformed by PITA, we note that the latter method uses secondary structure of the target to perform the predictions while the proposed method uses only the sequence. We observe that HuMiTar obtains higher TP rates when assuming moderate values of FP rates, i.e., it correctly predicts more functional duplexes when assuming a larger number of false positives. When using the default values of the scoring threshold, which equals 70, the proposed method obtains TP rate = 0.85 and FP rate = 0.44. This shows that HuMiTar predicts significant majority of the actual duplexes while achieving acceptable FP rate when compared with the other considered methods at this TP rate. The only method that obtains such high TP rate is PITA and its corresponding FP rate equals 0.41 (for both versions with and without flanking nucleotides).

### Predictions on p53

HuMiTar was applied to predict targets on p53, which is one of the most important tumor suppressor proteins. We note that PicTar does not report predictions for this target. Our method predicted total of 147 miRs that target this protein, and 15 of them, see Table 2, coincide with microarray-based results in [31]. We are currently unable to confirm or refute the remaining predictions.

**Table 2:** List of 15 miRs that were predicted by HuMiTar to target p53 and which were confirmed by Xi and colleagues (Xi et al., 2006).

hsa-let-7a	hsa-miR-296	hsa-miR-125b	hsa-miR-183	hsa-miR-19b
hsa-miR-30b	hsa-miR-30c	hsa-miR-30a-5p	hsa-miR-30d	hsa-miR-27a
hsa-miR-103	hsa-miR-107	hsa-miR-92	hsa-miR-10a	hsa-miR-326

### Genome-wide target prediction

Table 3 shows an overview of predictions on 39,215 3'UTR sequences in human genome and on 15 other genomes. The table shows the number of miRs used to predict sites for each species, the total number of targets predicted by HuMiTar, and the average number of predicted targets per one miR. Our predictions indicate that, on average, the number of targets for a single miR across a genome equals 9,613. We also observe that the number of predicted targets per miR is similar between different genomes.

**Table 3:** Summary of genome-wide predictions with HuMiTar.

Species type	# of miRs used	Total # of targets predicted with HuMitar	Average number of targets per miR
Anopheles gambiae	37	324050	8758

Bos taurus	125	1234929	9879

Caenorhabditis briggsae	93	759339	8165

Caenorhabditis elegan	134	1035742	7729

Drosophila melanogaster	78	728121	9335

Drosophila pseudoobscura	68	653553	9611

Fugu rubripes	109	1083787	9943

Gallus gallus	133	1302995	9797

Homo sapiens	471	4591244	9748

Macaca mulatta	70	738781	10554

Monodelphis domestica	100	1038582	10386

Mus musculus	380	3470973	9134

Pan troglodytes	80	843686	10546

Rattus norvegicus	238	2379080	9996

Tetraodon nigroviridis	109	1090377	10003

Xenopus tropicalis	160	1634721	10217

average	*149*	*1431873*	*9613*

One of the accepted ways of assessing the statistical significance of predicted targets, which was performed in [3,10,11], is based on using random miR sequences ('mock' miRs) as controls [17]. The motivation is that the mock sequences are unlikely to be biologically relevant and thus observing the ratio between the number of predictions for real miRs and for the mock miRs would indicate how many of the predictions for real miRs are indeed biologically relevant. The ratio of 'real' versus 'mock' predictions is provided as an estimate of the signal-to-noise ratio (SNR) of the target predictions [17]. The HuMiTar's SNR was evaluated using the set of 58 miRs and the randomization procedure to generate the mock miRs that were originally used to estimate SNR for PicTar [10]. The SNR of HuMiTar for the 58 real/mock miRs in the entire human 3'UTR set equals 1.99. PicTar's SNR was estimated to equal 1.8 when considering conservation using human, chimpanzee and mouse genomes, and 2.3 and 3.6 when dog and chicken genomes were added, respectively [10]. In case of TargetScanS the ratio was estimated to be 2.4 and 3.8 when considering all predictions and when considering only the positions conserved in all five genomes, respectively [11]. We note that the latter improved SNR was also accompanied by a 51% loss in sensitivity [11]. The SNR of the proposed method, which does not implement cross-species conservation, is comparable with the ratio of PicTar that was computed when the cross-species conservation was limited to human, chimpanzee and mouse genomes, and to the SNR of TargetScanS when conservation was not included. We anticipate that the SNR would increase if we would incorporate the cross-species conservation to filter our predictions.

### Computational complexity

The asymptotic computational complexity of HuMiTar is *O*(*m*^2^*n*) where *m *is the length of miR sequence and *n *is the length of the mRNA. This dominant factor contributing to the overall complexity is computation of 2D-coding that is used to perform initial screening of the mRNA. We note that since *m *is a small constant, the proposed method is characterized by a linear complexity with respect to the length of the mRNA sequence. We also performed experimental evaluation of the execution time. Using the set of 39,215 human 3'UTR sequences (the total lengths of these sequences equals 3.62e+07) the search for miR-21 targets takes 1,488 seconds using a desktop computer. We also computed execution times for ten randomly drawn miRs shown in Table 9 [see Additional file 1]. The targets were predicted on average in 1,451 seconds (about 24 minutes) per one miR, which shows that the proposed method can be applied on the genomic scale.

## Conclusion

The prediction of animal miR targets is an open and difficult problem in spite of several years of the existing research. HuMiTar, which is a prediction method designed based on human miR targets, is shown to provide predictions characterized by favorable sensitivity, which comes at a price of an increased number of predictions. The HuMiTar's predictions cover majority of the predictions of the best-performing competing methods such as PicTar, TargetScanS, and NBmiRTar. HuMiTar has good computational efficiency and comparable signal-to-noise ratio when compared with TargetScanS and PicTar. ROC analysis shows that HuMiTar provides predictions of quality that is comparable with the quality of PicTar, while our predictions are characterized by high true positive rates and moderate values of false positive rates. Our prediction method constitutes a step towards providing an efficient computational model for studying translational gene regulation by microRNAs. Our future work will concentrate of relaxation of the base-pairing requirements in the seed region to accommodate for miR-mRNA with the imperfect pairing and inclusion of information based on the stacked pairs and unpaired regions.

## Methods

HuMiTar works in two steps: (1) a 2D-coding method finds candidate targets by scanning 3'UTR of a given mRNA; and (2) the selected candidate targets are filtered using a composite scoring function.

### Scoring function

The scoring function was designed based on statistical analysis of 66 miR-mRNAs duplexes from the design set. Based on analysis of several recent studies [2-14,24], the miR sequence was divided into four regions: (1) position 1; (2) seed region (positions 2 to 8); (3) region 1 (positions 9 to 13); and (4) region 2 (positions 14 to 20). We computed conditional (assuming that they concern only the actual sites) and unconditional (assuming any other position in the mRNA using a sliding window) frequencies of the nucleotide pairs formed between miR's seed region, region 1, and region 2 and the corresponding 3' UTR of the mRNA. These values were used to compute affinity of each pair to form a bond between miR and mRNA; the affinity values are shown in Table 11 [see Additional file 1]. The differences in the obtained affinity values of the same pairs for different regions show that the occurrence of the corresponding pairs in a considered candidate complex should be weighed accordingly. We apply the principles of balance of moments to compute the weight values. The underlying interpretation is that the high affinity to bind in the seed region between a given miR and mRNA fragment should be balanced by sufficiently large affinity to bind in the non-seed regions (regions 1 and 2). We assume that the sum of moments generated by positions in the seed region should be greater than the sum of moment of the positions in non-seed regions. This problem is formulated and solved, i.e., the corresponding scoring function that optimizes the balance between binding in the seed and the non-seed regions is parameterized, using a standard linear programming model. The parameterization shows that the formation of complementary pairs for positions 9, 10, 12, 15, 16, 19 and 20 is less "important" than for the positions 11, 13, 14, 17 and 18. This result can be validated against a recent study that investigated Watson-Crick pairing for contiguous nucleotides. This study concluded that when excluding the seed, positions 13–16 have the strongest preference for the complementary pairing [37]. Although we consider each position individually, while the other study analyzed multimers, we also found that positions 13 and 14 have strong tendency to form complementary pairs. Our prediction model considers existence of two opposing forces: positive effect corresponding to the formation of complementary base pairs (which is quantified by the *reward function*), and negative effect due to the existence of non-complementary base pairs (quantified by the *penalty function*). The scoring function is defined as a difference between the values produced by the reward and the cost functions.

### 2D-coding method

The 3' UTR of mRNA is scanned using a sliding window. The basic principle of the 2D-coding is to scan an mRNA segment starting at a given position (using the sliding window) by finding stretches (segments) of complementary base pairs, which are denoted by *A*_*i *_where *i *= 1, 2, ..., 5. We start with finding the first segment, denoted by *A*_1_, in the miR's seed region, and then continue along the miR's sequence to find the subsequent segments. Given that a sufficient number of such segments can be found, which depends on several parameters explained in the [Additional file 1], the 2D-coding will output a candidate complex. The procedure will stop after finding *A*_5 _since no more then five complementary segments were found when scanning duplexes in the design set.

**Figure 5:**
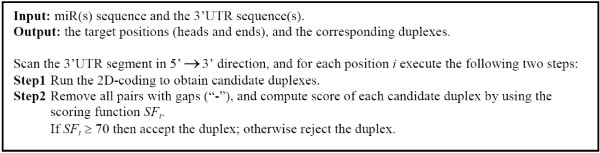
**Pseudo-code of the MuMiTar algorithm**.

The high-level pseudo-code of HuMiTar method is shown in Figure 5. Detailed description of the algorithm including pseudo-code of the 2D-coding method can be found in the [Additional file 1].

## Competing interests

The authors declare that they have no competing interests.

## Authors' contributions

JR contributed to the conception and the design of the project and the prediction method, contributed to the design of the experimental study and evaluation of the results, drafted and corrected the original and the revised manuscripts, and coordinated the project. HC contributed to the design of the prediction method and the experimental study, helped with the preparation of the datasets, performed computations, generated the prediction model and the experimental results, and helped with evaluation of the results. LK contributed to the design of the prediction method and the experimental study, evaluation of the results, drafted and corrected the original and the revised manuscripts, prepared the revisions, and coordinated the project. KC contributed to the design of the project and evaluation of the results. CK and PP contributed to the conception and the design of the project, helped with the design of the experimental study and evaluation of the results, and performed and interpreted Western blots. All authors read and approved all versions of the manuscript.

## Supplementary Material

Additional File 1 **Supplement for article entitled "HuMiTar: A sequence-based method for prediction of human microRNA targets"**. The file provides detailed description of the HuMiTar algorithm, 13 supplementary tables that give detailed test results and information that supports the detailed description of the algorithm, and 4 supplementary figures that assist in explaining the algorithm. Click here for file
