# Supplementary material for: HuMiTar: A sequence-based method for prediction of human microRNA targets
Source: Algorithms Mol Biol. 2008 Dec 22;3:16. doi: 10.1186/1748-7188-3-16 (PMC2631598; doi:10.1186/1748-7188-3-16)
Supplement: Additional File 1 — Supplement for article entitled "HuMiTar: A sequence-based method for prediction of human microRNA targets". The file provides detailed description of the HuMiTar algorithm, 13 supplementary tables that give detailed test results and information that supports the detailed description of the algorithm, and 4 supplementary figures that assist in explaining the algorithm. [file 1748-7188-3-16-S1.pdf]

Supplement for article entitled "HuMiTar: A sequence-based method for prediction of human microRNA targets"

Jishou Ruan[1], Hanzhe Chen[1], Lukasz Kurgan[2*], Ke Chen[2], Chunsheng Kang[3] and Peiyu Pu[3]

[1]Chern Institute for Mathematics, College of Mathematics and LPMC, Nankai University, Tianjin, PRC

[2]Department of Electrical and Computer Engineering, University of Alberta, Canada

[3]Neuro-oncology laboratory, General Hospital of the Tianjin Medical University, Tianjin, PRC

[*]Author to whom correspondence should be addressed.
Department of Electrical and Computer Engineering, University of Alberta, Canada, T6G 2V4, phone (780) 4925488, fax (780) 4921811, email: lkurgan@ece.ualberta.ca

Detailed description of HuMiTar

HuMiTar works in two steps: (1) a 2D-coding method finds candidate targets by scanning 3'UTR of a given mRNA; and (2) the selected candidate targets are filtered using a composite scoring function.

Motivation

A number of recent contributions discuss characteristic features of the miR-mRNAs duplexes [1-14]. They usually subdivide miR sequence into four regions. Although this division seems to be consistent between different works, their conclusions with respect to the formation of complementary base-pairing in these regions vary. One explanation for these differences is that the conclusions were based on different and limited size data. We summarize potential configurations of miR-mRNA duplexes as follows:

- In position 1, the pair may or not be complementary.
- In seed region (positions 2 to 8), the base pairing is usually assumed to be perfectly complementary [1-13]. The G:U (G:T) pairing is not permitted in seed region but it is allowed in the remaining positions.
- In region 1 (positions 9 to 13), the complementarity of the base pairs was investigated and assumed important in only a few contributions [1, 2, 6, 13], suggesting that their formation could have limited impact on the formation of the duplexes.
- In region 2 (positions 14 to 20), partially complementary base pairing is formed [1, 2, 4-8]; the complementarity is found to be important, but it is not required for all positions, as in the case of the seed region.

These results, which indicate the importance of base pairing of regions outside of the seed, motivate development of the proposed method. Since the conclusions concerning complementarity for regions 1 and 2 were based on samples of limited size (the abovementioned contributions studied only a few duplexes), we computed statistical information that aims at confirming/refuting these observations using the design set of 66 experimentally derived human miR-mRNAs duplexes shown in Table 1.

We use this information to parameterize the proposed prediction method, i.e., to establish weights that quantify the degree to which each of the positions in the miR sequence is required to form complementary pairs. The weights are used to develop reward and penalty functions, which together are used to implement scoring function that is applied to filter potential miR-mRNA duplexes. Although the length of the miRs can range between 18 and 28 nts, we decided to fix it at 21 nts, since majority of the miR have at least 21 nts. This allows assuring that enough statistical information is used to estimate the weights for these positions, i.e., number of duplexes with miRs longer than 21 would be too small to get a good estimate for weight values.

We note that inclusion of the stacked pairs and information concerning unpaired regions could lead to improved prediction rates. Due to limited sample size (number of duplexes used to parameterize the scoring function), inclusion of these factors could lead to the prediction model that would not generalize well outside of the training duplexes. We plan to include these factors, as the future work, when the amount of available training/test duplexes will increase.

Statistical analysis of base pairing in the miR-mRNA complex

First, we concentrate on the analysis of the distribution of potential base pairs in the seed region. The conditional frequencies of the potential nucleotide pairs formed between miR's seed region and the corresponding mRNA site, $p(T_i{:}T_j\,|\text{mRNA site})$, where T_i and T_j $\in$ {A, C, G, T(U)} and given that the binding concerns the actual site, are shown in Table 10. As expected, the complimentary C: G and A: T (U) pairs dominate the binding; the other pairs combined amount to only about 5% of cases. Although the remaining 5% of pairs agree with recent the results that suggest that imperfect pairing in the seed region could occur [14], the results clearly indicate that Watson-Crick base pairing is dominant in this region. The unconditional frequencies, $q(T_i{:}T_j)$, which are defined as the frequency of the base pairs $T_i{:}T_j$ computed by sliding the miR's seed region over all 7 nts drawn from the 66 mRNAs are shown in Table 10. The affinity of each nucleotide pair to form a bond between miR and mRNA is defined as $k(T_i{:}T_j) = \log_2(p(T_i{:}T_j\,|\text{mRNA site})/q(T_i{:}T_j))$, see Table 11. Although $p(A{:}G)$ and $p(T(U){:}C)$ equal 0, since our data is limited to 66 sites we anticipate that these pairs could occur in the miR-mRNA duplex with a low probability, i.e., $p(A{:}G) = 2^{-10}\ q(A{:}G)$, and thus the corresponding affinities of A:G and C:T(U) pairs are assumed to equal -10. The same computations were performed for miR sequence located in regions 1 and 2 combined, see Table 11.

The affinity values in the seed and non-seed regions allow to quantify the relative differences in binding of individual nucleotide pairs (include the Watson-Crick and other pairings). The differences in affinity values of the same pairs for different regions show that the existence of the corresponding pairs in a considered candidate complex should be weighed accordingly. We apply the principles of balance of moments, in which each pair at position k is characterized by the mass $k(T_i{:}T_j)$ (which corresponds to the affinity to form bonds in the miR-mRNA complexes) and arm length x_k, and where the moment value is computed as $k(T_i{:}T_j)\times x_k$. The underlying interpretation is that the high affinity to bind in the seed region between a given miR and mRNA fragment should be balanced by sufficiently large affinity to bind in the non-seed regions (regions 1 and 2). At the same time, the affinity is positively affected by the formation of complementary base pairs (which is quantified by the *reward function*), and negatively affected by formation of non-complementary base pairs (which is quantified by the *penalty function*). The strength of the impact of individual nucleotide pairs is estimated using the affinity coefficients $k(T_i{:}T_j)$ shown in Table 11. We assume that the sum of moments generated by positions in the seed region should be greater than the sum of moment of the positions in regions 1 and 2. This problem is formulated and solved, i.e., the corresponding scoring function that optimizes the balance between binding in the seed and the non-seed regions is parameterized, using a standard linear programming model.

Reward function

The reward function computes a score based on weighted sum of binding affinity coefficients for the complementary C:G and A:T(U) pairs (where different weights are used for different regions, see Table 11) along all positions in the seed region and the regions 1 and 2. Our approach balances the impact of complementary pairs in the seed region and with the complementary pairs in the non-seed region.

Assuming that the arm length value for the complementary pairs in the seed regions are assumed to equal 10 (all positions in the seed region are assumed equally important as

they all usually include complementary base pairs), the two moments are defined as

$$S(G:C)=\sum_{k=2}^{8}10\times 1_{\{G:C;\,C:G\}}(X_k:Y_k)\log_2\frac{p(G:C)}{q(G:C)}$$

$$S(A:U)=\sum_{k=2}^{8}10\times 1_{\{A:U;\,U:A\}}(X_k:Y_k)\log_2\frac{p(A:U)}{q(A:U)}$$

where p and q denote the conditional and unconditional frequencies of nucleotide pairs, respectively, and $S(G:C)$ and $S(A:U)$ applies to seed positions where the G:C and A:T(U) pairs are identified, correspondingly. The sum of $S(G:C)$ and $S(A:U)$ moments is considered as the *total moment of the seed region*. The minimal total moment value when complementary binding is assumed for the seed region equals $10*6*k(A:U)$, in which case positions 2 to 7 include A:U base pairs and positions 1 and 8 include non-complementary pairs.

The *total moment of the non-seed region* is defined as

$$S(3')=\sum_{k=1}^{12}x_k 1_{\{(A:U),(U:A),(G:C),(C:G),(G:U),(U:G)\}}(X_{8+k}:Y_{8+k})\log_2\frac{p(X_{8+k}:Y_{8+k})}{q(X_{8+k}:Y_{8+k})}$$

where x_k is the arm length of kth position, k=9,10, …,20, which values are estimated below, and where G:T (U) pairing is permitted. Assuming that the total moment (sum of the moments) for positions within the non-seed region should be smaller than the minimal total moment for the seed region, the arm length values used to implement the moment of the non-seed region should satisfy the following

$$\begin{cases}\max\left\{\sum_{k=1}^{12}x_k 1_{\{(A:U),(U:A),(G:C),(C:G),(G:U),(U:G)\}}(X_{8+k}:Y_{8+k})\log_2\frac{p(X_{8+k}:Y_{8+k})}{q(X_{8+k}:Y_{8+k})}\right\}\le 6\times 10\times k(A:U)\\ x_1,x_2,\dots,x_{12}\ge 0\end{cases}$$

I

n other words,

$$\begin{cases}Y\left(x_1,\dots,x_{12}\right)^{\tau}\le(\overbrace{b,\dots,b}^{N})^{\tau}\\ x_1,x_2,\dots,x_{12}\ge 0\end{cases}$$

in which

$$Y=\begin{pmatrix}1_{\mathsf{A}}(X_9^1:Y_9^1)\log_2\frac{p(X_9^1:Y_9^1)}{q(X_9^1:Y_9^1)} & \cdots\ \cdots & 1_{\mathsf{A}}(X_{20}^1:Y_{20}^1)\log_2\frac{p(X_{20}^1:Y_{20}^1)}{q(X_{20}^1:Y_{20}^1)}\\ 1_{\mathsf{A}}(X_9^2:Y_9^2)\log_2\frac{p(X_9^2:Y_9^2)}{q(X_9^2:Y_9^2)} & \cdots\ \cdots & 1_{\mathsf{A}}(X_{20}^2:Y_{20}^2)\log_2\frac{p(X_{20}^2:Y_{20}^2)}{q(X_{20}^2:Y_{20}^2)}\\ \cdots & \cdots\ \cdots & \cdots\\ 1_{\mathsf{A}}(X_9^N:Y_9^N)\log_2\frac{p(X_9^N:Y_9^N)}{q(X_9^N:Y_9^N)} & \cdots\ \cdots & 1_{\mathsf{A}}(X_{20}^N:Y_{20}^N)\log_2\frac{p(X_{20}^N:Y_{20}^N)}{q(X_{20}^N:Y_{20}^N)}\end{pmatrix}$$

$$=(Y_1,Y_2,\dots,Y_{12})$$

and

$b = 10*6*k(A:U)$, $\mathsf{A} = \{(A:U), (U:A), (G:C), (C:G), (G:U), (U:G)\}$ and $N = 12$.

The above boils down to solving the below linear programming problem

$$\min\left\{E(x_1Y_1 + x_2Y_2 + \cdots x_{12}Y_{12})\right\}$$

with the following solution

$(x_1, x_2, \ldots, x_{12})$=(3.89578, 4.53040, 12.50749, 2.33966, 12.87955, 23.26832, 1.69679, 2.97427, 9.75416, 13.72082, 0, 3.67476)

The solution shows that the formation of complementary pairs for positions 9, 10, 12, 15, 16, 19 and 20 is less "important" (has smaller arm length values) than for the positions 11, 13, 14, 17 and 18. We note that a recent study that investigated Watson-Crick pairing for contiguous nucleotides concluded that positions 13-16 have the strongest preference for the complementary pairing [15]. Although we consider each position individually, while the other study analyzed multimers, we observe certain similarities. In both cases, positions 13 and 14 are considered to have stronger tendency to form complementary pairs.

Finally, the *reward function* is defined as

$$R = S(G{:}\ C) + S(A{:}\ U) + S(3')$$

An empirical test with the design dataset shows that the reward function, which is based solely on formation of complementary pairs along the entire miR sequence, is not sufficient to distinguish between true and false targets. Figure 1A shows a distribution of the reward score values for targets that exclude the actual binding sites, while Figure 1B shows the distribution for the actual targets. The reward scores of the 66 miR-mRNA targets range between 133.9 and 245, while the scores of a set of non miR-mRNA targets range between 9.6 and 208.9. Although the overlap between the reward scores for the actual and the false sites is relatively small when compared with the overall range of values, see Figure 1, it does not allow perfect separation of the targets. As a result, we introduce the penalty function that quantifies a penalty for all non-complementary pairs formed with a given target.

Penalty function

The cost function is defined as:

$$C_i = 10\sum_{k=2}^{8} 1_{\mathrm{M}}\,(T_k : m_{i+21-k})\log_2\frac{p(T_k : m_{i+21-k})}{q(T_k : m_{i+21-k})} + \sum_{k=9}^{21} x_k 1_{\mathrm{M}}\,(T_k : m_{i+21-k})\log_2\frac{p(T_k : m_{i+21-k})}{q(T_k : m_{i+21-k})}$$

where $T_1T_2 \ldots T_{21}$ denotes a miR sequence, $m_N\, m_{N\text{-}1} \ldots m_2\, m_1$ denotes an inversely ordered segment of an mRNA sequence, $1_{\mathrm{M}}\,(T_k : m_{i+21-k})$ indicates a given nucleotide pair in which M is a set of non-complementary pairs (A:A, G:G, etc.), and the values of p and q are shown in Table 10.

Scoring function

The scoring function is defined as a difference between the reward and the cost functions:

$$SF_i = R - \mathrm{C}_i$$

where i denotes the target's position in the mRNA sequence.

Figure 2A shows a distribution of the scoring function values for targets that exclude the actual binding sites, while Figure 2B shows the distribution for the actual targets. We observe that the separation between the set of scores for actual miR-mRNA duplexes and false targets is improved when compared with using the reward function alone; compare Figures 1 and 2. Most specifically, the false targets generate scores between -512.9 and

150.6, while the scores for the true targets range between 12.8 and 243.4. Using a threshold value equal to 70, there are only 7 false miR-mRNA duplexes (duplexes that involve some miRs from the design set that target positions which are not published in TarBase) in the interval (70,150.6), and only 4 true miR-mRNA sites in the interval (12.8, 70). Therefore, using this threshold on the design dataset, i.e., we assume that a given predicted miR-mRNA duplex is true if the corresponding $SF_i \geq 70$, results in generating 7 false positives and 4 false negatives.

2D-coding method

The actual miR-mRNA duplexes may involve more than 21nts due to the formation of bulges. Within the design dataset that includes 66 actual miR-mRNA duplexes, the maximal length of the corresponding mRNA sequence is 46nts, while the maximal miR's length is 25nts. For example, the miR-mRNA duplex shown in Figure 3 includes 25nts for miR and 23nts for mRNA. This duplex can be rewritten in a linear form as follows

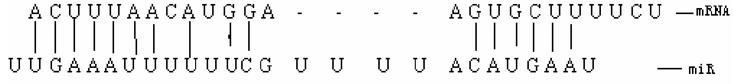

After removing the non-matching bulge segment

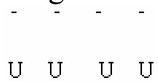

the corresponding *compacted form* of above duplex is

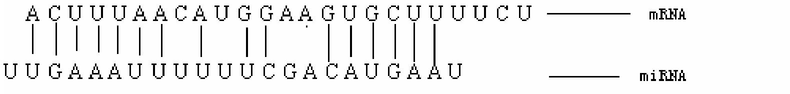

We use the compacted form to compute the scoring function value.

Following this example, we introduce a 2D-coding method that aims at generation of the compacted duplex form. Assuming that $T_1T_2 \ldots T_{21}$ denotes a miR and $m_N m_{N\text{-}1} \ldots m_2 m_1$ denotes the inversely ordered segment of an mRNA sequence we consider the following duplex

$$m_N, m_{N\text{-}1}, \ldots, m_2, m_1$$
$$T_1T_2 \ldots T_{21}$$

The basic principle of the 2D-coding is to scan an mRNA segment by finding stretches (segments) of complementary base pairs, which are denoted by A_i where $i = 1, 2, \ldots, 5$. We start with finding the first segment, denoted by A_1, in the miR's seed region, and then continue along the miR's sequence, see Figure 4.

The procedure will stop after finding A_5 since no more then five complementary segments can be found for the considered duplexes in the design set. The 2D-coding converts the original m_N $m_{N\text{-}1} \ldots m_2$ m_1 and $T_1T_2 \ldots T_k$ ($k \leq 25$) sequences into their corresponding compacted forms. The compacted form uses $\{a, c, g, u, A_1, A_2, A_3, A_4, A_5\}$ alphabet where a, c, g, and u denote non-complementary pairs and A_i denotes the complementary segments. The 2D-coding algorithm applies two thresholds, which are equal 15 (in steps II and VI) and 47 (in steps V and VII). The former threshold specifies the distance between A_1 and A_2, which is also used in [7]. The threshold value was computed as a sum of the average distance between A_1 and A_2 (7.83) and the standard deviation of the average (7.75) over the human miR-mRNA duplexes. The second threshold was computed as 26+3*7 = 47 where 26 and 7 are the average length and standard deviation of mRNA segments in the human miR-mRNA duplexes, respectively.

Using the proposed 2D-coding method, the miR and corresponding mRNA sequences in the compacted form may have different length. They can be aligned based on the scoring matrix shown in Table 12. Table 13 shows several example compacted forms that were obtained using the 2D-coding method.

HuMiTar algorithm

The pseudo-code of HuMiTar method is shown in Figure 5 in the main text.

References

1. Enright AJ, John B, Gaul U, Tuschl T, Sander C, Marks DS: **MicroRNA targets in Drosophila**. *Genome Biol.* 2003, **5**(1):R1.
2. Lewis BP, Shih I, Jones-Rhoades MW, Bartel DP, Burge CB: **Prediction of mammalian microRNA targets**. *Cell* 2003, **115**:787–798.
3. Stark A, Brennecke J, Russell RB, Cohen SM: **Identification of Drosophila MicroRNA targets**. *PLoS Biol.* 2003, **1**(3):e397.
4. Doench JG, Sharp PA: **Specificity of microRNA target selection in translational repression**. *Genes Dev.* 2004, **18**(5):504-511.
5. John B, Enright AJ, Aravin A, Tuschl T, Sander C, Marks DS: Human **microRNA targets**. *PLoS Biol.* 2004, **2**(11):e363.
6. Kiriakidou M, Nelson P, Kouranov A, Fitziev P, Bouyioukos C, Mourelatos Z, Hatzigeorgiou AG: **A combined computational- experimental approach predicts human miR targets**. *Genes & Dev.* 2004, **18**:1165–1178.
7. Rehmsmeier M, Steffen P, Hochsmann M, Giegerich R: **Fast and effective prediction of microRNA/target duplexes**. *RNA* 2004, **10**:1507–1517.
8. Vella MC, Reinert K, Slack FJ: **Architecture of a validated microRNA: target interaction**. *Chem. Biol.* 2004, **11**:1619–1623.
9. Krek A, Grun D, Poy MN, Wolf R, Rosenberg L, Epstein EJ, MacMenamin P, Piedade I, Gunsalus KC, Stoffel M, et al.: **Combinatorial microRNA target predictions**. *Nat. Genet.* 2005, **37**:495–500.
10. Lewis BP, Burge CB, Bartel DP: **Conserved seed pairing, often flanked by adenosines, indicates that thousands of human genes are microRNA targets**. *Cell* 2005, **120**:15–20.
11. Saetrom O, Snove O Jr, Saetrom P. **Weighted sequence motifs as an improved seeding step in microRNA target prediction algorithms**. *RNA* 2005, **11**(7): 995-1003.
12. Xie X, Lu J, Kulbokas EJ, Golub TR, Mootha V, Lindblad-Toh K, Lander ES, Kellis M: **Systematic discovery of regulatory motifs in human promoters and 3'UTRs by comparison of several mammals**. *Nature* 2005, **434**: 338–345.
13. Watanabe Y, Yachie N, Numata K, Saito R, Kanai A, Tomita M: **Computational analysis of microRNA targets in Caenorhabditis elegans**. *Gene* 2006, **365**:2-10.
14. Didiano D, Hobert O: **Perfect seed pairing is not a generally reliable predictor for miRNA-target interactions**. *Nat Struct Mol Biol.* 2006, **13**(9):849-51.
15. Grimson A, Kai-How Farth K, Johnston WK, Garrnet-Engele P, Lim LP, Bartel DP: **MicroRNA targeting specificity in mammals: determinants beyond seed pairing**, *Molecular Cell* 2007, **27**:91-105.

Supplementary tables

TABLE 1. The design dataset of 66 human miR-mRNA duplexes.

miR	Gene	# targets	miR	Gene	# targets
let-7a	KRAS2	5	miR-16	BCL2	1
let-7a	NRAS	4	miR-17-5p	E2F1	2
let-7b	Lin28	1	miR-199b	LAMC2	1
let-7e	SMC1L1	1	miR-19a	PTEN	1
miR-1	Hand2	1	miR-1b	G6PD	3
miR-1	TMSB4X	1	miR-1b	BDNF	3
miR-1	HDAC4	2	miR-20a	E2F1	2
miR-101	EZH2	2	miR-221	KIT	1
miR-101	MYCN	2	miR-222	KIT	1
miR-103	FBXW1B	1	miR-223	NFIA	1
miR-10a	HOXA1	1	miR-23	HES1'(Y07572)	1
miR-130	CSF1	1	miR-23	HES1(NM_005524)	3
miR-132	RICS (p250GAP)	1	miR-23	POU4F2	3
miR-133a	SRF	2	miR-23a	C6orf134	1
miR-141	Clock	1	miR-23a	CXCL12	2
miR-143	MAPK7	1	miR-24	MAPK14	1
miR-145	FLJ21308	1	miR-26	SMAD1	2
miR-15a	DMTF1	1	miR-34	DLL1	3
miR-15a	BCL2	1	miR-34	Notch1	2
miR-16	CGI-38	1	miR-375	Mtpn	1

TABLE 2. Oncogenes predicted by HuMiTar, PicTar, TargetScanS, and NBmiRTar.

Gene name	Gene ID	Description in homo sapiens
Cx43	NM_000165	gap junction protein, alpha 1, 43kDa (connexin 43)
KRAS2	NM_033360	v-Ki-ras2 Kirsten rat sarcoma viral oncogene homolog
EGFR	NM_005228	epidermal growth factor receptor oncogene homolog
CCND1	NM_053056	cyclin D1
WNT5A	NM_003392	wingless-type MMTV integration site family, member 5A
MYC	NM_002467	v-myc myelocytomatosis viral oncogene homolog
NOTCH1	NM_017617	notch homolog 1, translocation-associated
CTNNB1	NM_001904	catenin (cadherin-associated protein), beta 1, 88kDa
SEPT7	NM_001788	septin7 (SEPT7), transcript variant 1, mRNA
PTEN	NM_000314	phosphatase and tensin homolog

TABLE 3. List of 328 human miRs that are associated with the selected ten oncogenes

hsa-let-7a	hsa-miR-191	hsa-miR-325	hsa-miR-494
hsa-let-7b	hsa-miR-191*	hsa-miR-326	hsa-miR-495
hsa-let-7c	hsa-miR-192	hsa-miR-328	hsa-miR-496
hsa-let-7d	hsa-miR-193a	hsa-miR-329	hsa-miR-497
hsa-let-7e	hsa-miR-193b	hsa-miR-33	hsa-miR-498
hsa-let-7f	hsa-miR-194	hsa-miR-330	hsa-miR-499
hsa-let-7g	hsa-miR-195	hsa-miR-331	hsa-miR-500
hsa-let-7i	hsa-miR-196a	hsa-miR-335	hsa-miR-501
hsa-miR-1	hsa-miR-196b	hsa-miR-337	hsa-miR-502
hsa-miR-100	hsa-miR-197	hsa-miR-338	hsa-miR-503
hsa-miR-101	hsa-miR-198	hsa-miR-339	hsa-miR-504
hsa-miR-103	hsa-miR-199a	hsa-miR-340	hsa-miR-505
hsa-miR-105	hsa-miR-199a*	hsa-miR-342	hsa-miR-506
hsa-miR-106a	hsa-miR-199b	hsa-miR-345	hsa-miR-507
hsa-miR-106b	hsa-miR-19a	hsa-miR-346	hsa-miR-508
hsa-miR-107	hsa-miR-19b	hsa-miR-34a	hsa-miR-509
hsa-miR-10a	hsa-miR-200a	hsa-miR-34b	hsa-miR-510
hsa-miR-10b	hsa-miR-200a*	hsa-miR-34c	hsa-miR-511
hsa-miR-122a	hsa-miR-200b	hsa-miR-361	hsa-miR-512-3p
hsa-miR-124a	hsa-miR-200c	hsa-miR-362	hsa-miR-512-5p
hsa-miR-125a	hsa-miR-202	hsa-miR-363	hsa-miR-513
hsa-miR-125b	hsa-miR-202*	hsa-miR-363*	hsa-miR-514
hsa-miR-126	hsa-miR-203	hsa-miR-365	hsa-miR-515-3p
hsa-miR-126*	hsa-miR-204	hsa-miR-367	hsa-miR-515-5p
hsa-miR-127	hsa-miR-205	hsa-miR-368	hsa-miR-516-3p
hsa-miR-128a	hsa-miR-206	hsa-miR-369-3p	hsa-miR-516-5p
hsa-miR-128b	hsa-miR-208	hsa-miR-369-5p	hsa-miR-517*
hsa-miR-129	hsa-miR-20a	hsa-miR-370	hsa-miR-517a
hsa-miR-130a	hsa-miR-20b	hsa-miR-371	hsa-miR-517b
hsa-miR-130b	hsa-miR-21	hsa-miR-372	hsa-miR-517c
hsa-miR-132	hsa-miR-210	hsa-miR-373	hsa-miR-518a
hsa-miR-133a	hsa-miR-211	hsa-miR-373*	hsa-miR-518a-2*
hsa-miR-133b	hsa-miR-212	hsa-miR-374	hsa-miR-518b
hsa-miR-134	hsa-miR-213	hsa-miR-375	hsa-miR-518c
hsa-miR-135a	hsa-miR-214	hsa-miR-376a	hsa-miR-518c*
hsa-miR-135b	hsa-miR-215	hsa-miR-376a*	hsa-miR-518d
hsa-miR-136	hsa-miR-216	hsa-miR-376b	hsa-miR-518e
hsa-miR-137	hsa-miR-217	hsa-miR-377	hsa-miR-518f
hsa-miR-138	hsa-miR-218	hsa-miR-378	hsa-miR-518f*
hsa-miR-139	hsa-miR-219	hsa-miR-379	hsa-miR-519a
hsa-miR-140	hsa-miR-22	hsa-miR-380-3p	hsa-miR-519b
hsa-miR-141	hsa-miR-220	hsa-miR-380-5p	hsa-miR-519c
hsa-miR-142-3p	hsa-miR-221	hsa-miR-381	hsa-miR-519d
hsa-miR-142-5p	hsa-miR-222	hsa-miR-382	hsa-miR-519e
hsa-miR-143	hsa-miR-223	hsa-miR-383	hsa-miR-519e*
hsa-miR-144	hsa-miR-224	hsa-miR-384	hsa-miR-520a
hsa-miR-145	hsa-miR-23a	hsa-miR-409-3p	hsa-miR-520a*
hsa-miR-146a	hsa-miR-23b	hsa-miR-409-5p	hsa-miR-520b
hsa-miR-146b	hsa-miR-24	hsa-miR-410	hsa-miR-520c
hsa-miR-147	hsa-miR-25	hsa-miR-412	hsa-miR-520d
hsa-miR-148a	hsa-miR-26a	hsa-miR-422a	hsa-miR-520d*
hsa-miR-148b	hsa-miR-26b	hsa-miR-422b	hsa-miR-520e
hsa-miR-149	hsa-miR-27a	hsa-miR-423	hsa-miR-520f
hsa-miR-150	hsa-miR-27b	hsa-miR-424	hsa-miR-520g

hsa-miR-151	hsa-miR-28	hsa-miR-425	hsa-miR-520h
hsa-miR-152	hsa-miR-296	hsa-miR-429	hsa-miR-521
hsa-miR-153	hsa-miR-299-3p	hsa-miR-431	hsa-miR-522
hsa-miR-154	hsa-miR-299-5p	hsa-miR-432	hsa-miR-523
hsa-miR-154*	hsa-miR-29a	hsa-miR-432*	hsa-miR-524
hsa-miR-155	hsa-miR-29b	hsa-miR-433	hsa-miR-524*
hsa-miR-15a	hsa-miR-29c	hsa-miR-448	hsa-miR-525
hsa-miR-15b	hsa-miR-301	hsa-miR-449	hsa-miR-525*
hsa-miR-16	hsa-miR-302a	hsa-miR-450	hsa-miR-526a
hsa-miR-17-3p	hsa-miR-302a*	hsa-miR-451	hsa-miR-526b
hsa-miR-17-5p	hsa-miR-302b	hsa-miR-452	hsa-miR-526b*
hsa-miR-181a	hsa-miR-302b*	hsa-miR-452*	hsa-miR-526c
hsa-miR-181b	hsa-miR-302c	hsa-miR-453	hsa-miR-527
hsa-miR-181c	hsa-miR-302c*	hsa-miR-455	hsa-miR-539
hsa-miR-181d	hsa-miR-302d	hsa-miR-483	hsa-miR-542-3p
hsa-miR-182	hsa-miR-30a-3p	hsa-miR-484	hsa-miR-542-5p
hsa-miR-182*	hsa-miR-30a-5p	hsa-miR-485-3p	hsa-miR-544
hsa-miR-183	hsa-miR-30b	hsa-miR-485-5p	hsa-miR-545
hsa-miR-184	hsa-miR-30c	hsa-miR-486	hsa-miR-7
hsa-miR-185	hsa-miR-30d	hsa-miR-487a	hsa-miR-9
hsa-miR-186	hsa-miR-30e-3p	hsa-miR-487b	hsa-miR-9*
hsa-miR-187	hsa-miR-30e-5p	hsa-miR-488	hsa-miR-92
hsa-miR-188	hsa-miR-31	hsa-miR-489	hsa-miR-93
hsa-miR-189	hsa-miR-32	hsa-miR-490	hsa-miR-95
hsa-miR-18a	hsa-miR-320	hsa-miR-491	hsa-miR-96
hsa-miR-18a*	hsa-miR-323	hsa-miR-492	hsa-miR-98
hsa-miR-18b	hsa-miR-324-3p	hsa-miR-493-3p	hsa-miR-99a
hsa-miR-190	hsa-miR-324-5p	hsa-miR-493-5p	hsa-miR-99b

TABLE 4. The prediction results for the design set of 66 human miR-mRNA duplexes.

[1] the top 51 duplexes include miRs with seed regions that are perfectly complementary to the corresponding coding regions; duplexes numbered 52 to 66 inclusive include miRs for which the coding region is only partially complementary to the coding region.
[2] the third column gives name of the 3'UTR of the corresponding target gene as listed in TarBase; since multiple 3'UTRs are possible for a given gene, we selected the longest 3'UTR that includes the target site.
[3] values the last five columns denote number of predicted targets; 1 means that the corresponding method correctly predicted a given target; 0 denotes the a given method failed to predict a given target; 1+*k* shows that the corresponding method predicted a given target as well as *k* extra, unpublished targets; 0+*k* means that the corresponding method failed to predict published target but predicted *k* extra unpublished targets.

no[1]	miR - mRNA pair	actual target[2] (start-end)	Predictions[3]				
			HuMiTar	PicTar	DIANA-MicroT	Target ScanS	NBmiRTar
1	miR-375 MTPN	NM_145808.1 (3121-3141)	1	1	0	1	1
2	let-7b LIN28	NM_024674.3 (890-912)	1	1+1	1	1	1+1
3	miR-141 Clock	NM_004898.2 (215-233)	1+2	0	1	1+1	0+1
4	miR-24 MAPK14	NM_001315.1 (651-669)	1	0	1	0	0
5	miR-23a C6orf134	NM_024909.1 (209-226)	1	0	1	1	0
6	let-7e SMC1L1	NM_006306.2 (73-91)	1+3	0	1	1+3	0+7
7	miR-15a DMTF1	NM_021145.1 (130-146)	1	1	1	1	1
8	miR-16 CGI-38	NM_016140.1 (294-311)	1+1	1	1	1	0
9	miR-199b LAMC2	NM_005562.1 (209-222)	1	0	1	0	1
10	miR-23 HES1	NM_005524.2 (267-288)	1	1	0	0	1
11	miR-20a E2F1	NM_005225.1 (371-394)	1	1	0	1	0
12	miR-20a E2F1	NM_005225.1 (943-987)	1	1	0	1	1+1
13	miR-17-5p E2F1	NM_005225.1 (371-394)	1	1+1	0	1	0
14	miR-17-5p E2F1	NM_005225.1 (943-987)	1	1	0	1	1+1
15	miR-143 MAPK7	NM_139032.1 (91-127)	1	0	0	1	1
16	miR-1 Hand2	NM_021973.1 (208-232)	1	1	0	1	0
17	miR-1 TMSB4X	NM_021109.2 (17-36)	1	0	0	1	1
18	miR-23 POU4F2	NM_004575.1 (89-109)	1	1	0	1	1
19	miR-23 POU4F2	NM_004575.1 (156-180)	1	1	0	0	1
20	miR-23 POU4F2	NM_004575.1 (449-470)	1	1	0	1	0
21	miR-101 EZH2	NM_004456.3 (46-66)	1	1	0	1	1
22	miR-101 EZH2	NM_004456.3 (88-121)	1	1	0	1	1
23	miR-101 MYCN	NM_005378.3 (579-501)	1	1	0	1	1
24	miR-101 MYCN	NM_005378.3 (553-570)	1	1	0	1	1
25	miR-19a PTEN	NM_000314.2 (396-418)	1	0	0	1	0
26	miR-34 DLL1	NM_005618.2 (183-204)	1	1	0	0	1
27	miR-34 DLL1	NM_005618.2 (281-300)	1	1	0	1	1
28	miR-34 DLL1	NM_005618.2 (333-363)	1	1	0	1	1
29	miR-34 Notch1	NM_017617.2 (145-186)	1+1	1+4	0	1	1+2

30	miR-34 Notch1	NM_017617.2 (894-916)	1	1	0	1	1
31	miR-1b G6PD	NM_000402.2 (83-104)	1	0	0	0	1
32	miR-1b G6PD	NM_000402.2 (140-172)	1	0	0	1	0
33	miR-1b G6PD	NM_000402.2 (419-440)	1	0	0	1	1
34	miR-1b BDNF	NM_170731.2 (194-227)	1	1+2	0	1	1
35	miR-1b BDNF	NM_170731.2 (375-395)	1	1	0	1	0
36	miR-1b BDNF	NM_170731.2 (1306-1329)	1	1	0	1	1
37	miR-130 CSF1	NM_000757.3 (782-807)	1	1+3	0	1	1
38	miR-26 SMAD1	NM_005900.1 (25-52)	1	1+1	0	1	0
39	miR-26 SMAD1	NM_005900.1 (91-109)	1	1	0	1	1
40	miR-23a CXCL12	NM_000609.3 (1352-1394)	1	0	0	0	0+3
41	miR-23a CXCL12	NM_000609.3 (1439-1459)	1	0	0	0	0
42	let-7a KRAS2	NM_033360.2 (3246-3273)	1	0	0	0	1
43	miR-15a BCL2	NM_000633.1 (2511-2536)	1+3	1+2	0	1	1
44	miR-16 BCL2	NM_000633.1 (2511-2536)	1+3	1+3	0	1	1
45	miR-132 RICS	NM_014715.2 (30-51)	1	1	0	1	0
46	miR-223 NFIA	NM_005595.1 (737-760)	1	1+1	1	1	1
47	miR-221 KIT	NM_000222.1 (1014-1037)	1+1	0	0	1	1
48	miR-222 KIT	NM_000222.1 (1014-1037)	1	0	0	1	1
49	miR-1 HDAC4	NM_006037.2 (3502-3522)	1	1+4	0	1	0
50	miR-1 HDAC4	NM_006037.2 (3534-3554)	1	1	0	1	1
51	miR-10a HOXA1	NM_153620.1 (947-976)	1	1+1	0	1	0
52	miR-145 FLJ21308	NM_024615.2	0	0	1	0	0
53	miR-103 FBXW1B	NM_012300.1	0+1	0	1	0	1+1
54	miR-23 HES1	Y07572	0	0	0	0	1
55	miR-23 HES1	NM_005524.2	0	0	0	0	0
56	miR-23 HES1	NM_005524.2	0	0	0	0	0
57	let-7a KRAS2	NM_033360.2	0	0	0	0	0+3
58	let-7a KRAS2	NM_033360.2	0	0	0	0	0
59	let-7a KRAS2	NM_033360.2	0	0	0	0	1
60	let-7a KRAS2	NM_033360.2	0	0	0	0	0
61	let-7a NRAS	NM_002524.2	0+1	0	0	0	0+1
62	let-7a NRAS	NM_002524.2	0	0	0	0	0
63	let-7a NRAS	NM_002524.2	0	0	0	0	0
64	let-7a NRAS	NM_002524.2	0	1	0	1	0
65	miR-133a SRF	NM_003131.1	0	0	0	0	1
66	miR-133a SRF	NM_003131.1	0	0	0	0	1
		total number of predicted published targets	51	36	11	43	38
		total number of predicted unpublished targets	16	23	0	4	21

TABLE 5. The prediction results for the independent set of 39 human miR-mRNA duplexes.

[1] the top 32 duplexes include miRs with seed regions that are perfectly complementary to the corresponding coding regions; duplexes numbered 33 to 39 inclusive include miRs for which the coding region is only partially complementary to the coding region.
[2] the third column gives name of the 3'UTR of the corresponding target gene as listed in TarBase; since multiple 3'UTRs are possible for a given gene, we selected the longest 3'UTR that includes the target site.
[3] values the last five columns denote number of predicted targets; 1 means that the corresponding method correctly predicted a given target; 0 denotes the a given method failed to predict a given target; 1+*k* shows that the corresponding method predicted a given target as well as *k* extra, unpublished targets; 0+*k* means that the corresponding method failed to predict published target but predicted *k* extra unpublished targets.

no[1]	miR - mRNA pair	actual target[2] (start-end)	predictions[4]				
			HuMiTar	PicTar	DIANA-MicroT	Target ScanS	NBmiR Tar
1	miR-155 AGTR1	NM_000685.3 (79-90)	1+2	0	0	1	0
2	miR-140 HDAC4	NM_006037.2 (439-460)	1	1+3	0	1	0
3	miR-17-5p NCOA3	NM_006534.2 (1282-1303)	1+4	1+3	0	1+1	1
4	miR-27b CYP1B1	NM_000104.2 (2726-2749)	1+1	1+5	0	1	0
5	miR-206 Fstl1	NM_007085.3 (2099-2121)	1+2	0	0	1+1	1
6	miR-206 Utrn	NM_007124.1 (454-477)	1	0	0	0	1
7	miR-189 SLITRK1	NM_052910.1 (675-697)	1	0	0	0	0
8	miR-206 GJA1	NM_000165.2 (459-485)	1+1	1+1	0	1	1+1
9	miR-206 GJA1	NM_000165.2 (1598-1618)	1	1	0	1	1
10	miR-1 GJA1	NM_000165.2 (467-485)	1+1	1+2	0	1	1+1
11	miR-1 GJA1	NM_000165.2 (1598-1618)	1	1	0	1	1
12	miR-29 Tcl1A	NM_021966.1 (428-450)	1	0	0	1	0
13	miR-122 SLC7A1	NM_003045.2 (1073-1098)	1+1	1	0	1	0+2
14	miR-122 SLC7A1	NM_003045.2 (1345-1371)	1	1	0	1	0
15	miR-125a ERBB2	NM_004448.1 (17-44)	1	0	0	1	1
16	miR-125b ERBB3	NM_001982.1 (8-26)	1	0	0	0	1
17	miR-133 PTBP2	NM_021190.1 (63-81)	1	1+1	1	1	0
18	miR-133 PTBP2	NM_021190.1 (1008-1042)	1	1	0	1	0
19	miR-34a E2F3	NM_001949.2 (2713-2736)	1	1+5	0	1	0+1
20	miR-21 TPM1	NM_000366.4 (221-242)	1	0	0	0	0
21	miR-376a-5p SFRS11	NM_004768.2 (761-783)	1+2	0	0	0	0
22	miR-376a-5p SFRS11	NM_004768.2 (924-948)	1	0	0	0	0
23	miR-376a-5p SLC16A1	NM_003051.2 (55-78)	1	0	0	0	0
24	miR-376a-5p SLC16A1	NM_003051.2 (812-833)	1	0	0	0	0
25	miR-376a-5p TTK	NM_003318.3 (54-81)	1	0	0	0	0
26	miR-376a-5p TTK	NM_003318.3 (208-229)	1	0	0	0	0
27	Edited-miR-376a-5p PRPS1	NM_002764.2 (17-39)	1	0	0	0	0
28	Edited-miR-376a-5p SNX19	NM_014758.1 (369-370)	1	0	0	0	0
29	Edited-miR-376a-5p SNX19	NM_014758.1 (687-704)	1	0	0	0	0

30	miR-208 THRAP1	NM_005121.1 (549-572)	1+1	1+1	0	1	0
31	miR-29b MCL1	NM_021960.3 (1318-1340)	1+1	1+1	0	1	0
32	miR-1 KCNJ2	NM_000891.2 (1062-1081)	1	0	0	0	1
33	miR-127 BCL6	NM_001706.2	0	0	0	0	0
34	miR-181 Tcl1A	NM_021966.1	0	0	0	0	0
35	miR-122 SLC7A1	NM_003045.2	0	1	0	0	0
36	let-7a NF2	NM_181826.1	0	0	0	0	0+1
37	Edited-miR-376a-5p PRPS1	NM_002764.2	0	0	0	0	0
38	Edited-miR-376a-5p ZNF513	NM_144631.4	0	0	0	0	0
39	Edited-miR-376a-5p ZNF513	NM_144631.4	0	0	0	0	0
		total number of predicted published targets	32	15	1	18	10
		total number of predicted unpublished targets	16	22	0	2	6

TABLE 6. Comparison of PicTar and HuMiTar predictions for GO set.

The reported values include the number of targets predicted by PicTar, the number of targets predicted by both PicTar and HuMiTar, the number of targets predicted only by PicTar, and the number of targets predicted only by HuMiTar.

[1]results in bold concern Septin7 for which experimental verification was performed

[2]results for PicTar are limited to a subset of miRs that were available in the PicTar's database (http://pictar.bio.nyu.edu/).

Gene ID	# targets predicted by PicTar[2]	# targets predicted by HuMiTar and PicTar	# targets predicted only by PicTar	# of targets predicted only by HuMiTar	
				For these miRs that were included in the PicTar's database	only for miRs that were not included in the PicTar's database
NM_000165	21	19	2	51	51
NM_033360	27	27	0	106	69
NM_005228	4	4	0	73	53
NM_053056	28	28	0	96	75
NM_003392	6	6	0	115	65
NM_002467	3	3	0	15	10
NM_017617	5	5	0	69	47
NM_001904	5	5	0	41	16
**NM_001788**[1]	**19**	**18**	**1**	**34**	**23**
NM_000314	14	13	1	46	33
Total	132	128 (97%)	4 (3%)	646	442

TABLE 7. Comparison of TargetScanS and HuMiTar predictions for GO set.

The reported values include the number of targets predicted by TargetScanS, the number of targets predicted by both TargetScanS and HuMiTar, the number of targets predicted only by TargetScanS, and the number of targets predicted only by HuMiTar.
[1]results in bold concern Septin7 for which experimental verification was performed.

Gene ID	# targets predicted by TargetScanS	# targets predicted by HuMiTar and TargetScanS	# targets predicted only by TargetScanS	# targets predicted only by HuMiTar
NM_000165	74	70	4	51
NM_033360	109	107	2	95
NM_005228	66	65	1	65
NM_053056	119	116	3	83
NM_003392	111	108	3	78
NM_002467	18	14	4	14
NM_017617	54	50	4	71
NM_001904	21	20	1	42
**NM_001788**[1]	**41**	**36**	**5**	**39**
NM_000314	109	68	41	24
Total	722	654 (91%)	68 (9%)	562

TABLE 8. Comparison of NBmiRTar and HuMiTar predictions for GO set.

The reported values include the number of targets predicted by NBmiRTar, the number of targets predicted by both NBmiRTar and HuMiTar, the number of targets predicted only by NBmiRTar, and the number of targets predicted only by HuMiTar.

Gene ID	# targets predicted by NBmiRTar	# targets predicted by HuMiTar and NBmiRTar	# targets predicted only by NBmiRTar	# targets predicted only by HuMiTar
NM_000165	45	23	22	98
NM_033360	88	65	23	137
NM_005228	34	14	20	116
NM_053056	91	70	21	129
NM_003392	78	34	44	152
NM_002467	6	0	6	28
NM_017617	63	30	33	91
NM_001904	22	10	12	52
NM_001788	28	10	18	65
NM_000314	25	11	14	81
Total	480	267 (56%)	213 (44%)	949

TABLE 9. List of 10 miRs used to calculate execution time.

hsa-miR-139	hsa-miR-106b	hsa-miR-21	hsa-miR-23a
hsa-miR-768-3p	hsa-miR-221	hsa-miR-222	
hsa-miR-15b	hsa-miR-27a	hsa-miR-23b	

TABLE 10. Conditional probability $p(T_i\!:\!T_j\,|\text{mRNA site})$ (top number) of nucleotide pairs from the seed regions of the 66 human miR-mRNAs duplexes, and unconditional probability, $q(T_i\!:\!T_j)$ (bottom number) of the binding of the miR's seed region along the entire 66 human mRNAs.

The matrix is symmetric, i.e., "-" denotes that the corresponding value is symmetric.

	A	C	G	T (U)
A	0.005<br>0.051	0.005<br>0.068	0<br>0.131	0.488<br>0.161
C	-	0.002<br>0.022	0.458<br>0.092	0<br>0.109
G	-	-	0.002<br>0.072	0.033<br>0.180
T (U)	-	-	-	0.<br>0.114

TABLE 11. miR-mRNA binding affinity $k(T_i\!:\!T_j)$ of nucleotide pairs from the seed region (top number) and from regions 1 and 2 combined (bottom number).

The matrix is symmetric, i.e., "-" denotes that the corresponding value is symmetric.

	A	C	G	T (U)
A	-3.37<br>-1.04	-3.78<br>-2.03	-10<br>-1.97	1.6<br>1.14
C	-	-3.74<br>-1.56	2.32<br>1.41	-10<br>-1.11
G	-	-	-5.44<br>-1.16	-2.43<br>0.31
T (U)	-	-	-	-4.1<br>-1.02

TABLE 12. Alignment matrix for compacted forms of miR-mRNA duplexes.

"–" denotes a gap, $|A_i|$ denotes length of string A_i, $s_{ij} = s_{ji} = -|A_i||A_j|$ where $i \neq j$.

	a	c	g	u	A_1	A_2	A_3	A_4	A_5	–
a	-2	-2	-2	2	$-\|A_1\|$	$-\|A_2\|$	$-\|A_3\|$	$-\|A_4\|$	$-\|A_5\|$	0
c	-2	-2	2	-2	$-\|A_1\|$	$-\|A_2\|$	$-\|A_3\|$	$-\|A_4\|$	$-\|A_5\|$	0
g	-2	2	-2	2	$-\|A_1\|$	$-\|A_2\|$	$-\|A_3\|$	$-\|A_4\|$	$-\|A_5\|$	0
u	2	-2	2	-2	$-\|A_1\|$	$-\|A_2\|$	$-\|A_3\|$	$-\|A_4\|$	$-\|A_5\|$	0
A_1	$-\|A_1\|$	$-\|A_1\|$	$-\|A_1\|$	$-\|A_1\|$	$-\|A_1\|^2$	s_{12}	s_{13}	s_{14}	s_{15}	$-\|A_1\|$
A_2	$-\|A_2\|$	$-\|A_2\|$	$-\|A_2\|$	$-\|A_2\|$	s_{21}	$-\|A_2\|^2$	s_{23}	s_{24}	s_{25}	$-\|A_2\|$
A_3	$-\|A_3\|$	$-\|A_3\|$	$-\|A_3\|$	$-\|A_3\|$	s_{31}	s_{32}	$-\|A_3\|^2$	s_{34}	s_{35}	$-\|A_3\|$
A_4	$-\|A_4\|$	$-\|A_4\|$	$-\|A_4\|$	$-\|A_4\|$	s_{41}	s_{42}	s_{43}	$-\|A_4\|^2$	s_{45}	$-\|A_4$
A_5	$-\|A_5\|$	$-\|A_5\|$	$-\|A_5\|$	$-\|A_5\|$	s_{51}	s_{52}	s_{53}	s_{54}	$-\|A_5\|^2$	$-\|A_5\|$
–	0	0	0	0	$-\|A_1\|$	$-\|A_2\|$	$-\|A_3\|$	$-\|A_4\|$	$-\|A_5\|$	-100

TABLE 13. Example compacted forms of miR-mRNA duplexes.

mRNA-miR	miR-mRNA duplex (mRNA at the top, miR below)	Compacted forms (mRNA at the top, miR below)
CX43-miR-30a-5p	UUUUUGUGGUGUGGGCCAAUAUGGUGUUUACA<br>•||• | •|•| |•||||||||| CGAAGGUCA--GCUC-------CUACAAAUGU	$uA_4guggugA_2ccaauauA_3uA_1a$<br>$cA_4gucaA_2A_3aA_1u$
CX43-miR-30d	UUUUGUGGUGUGGGCCAAUAUGGUGUUUACA<br>||• | •||| |•||||||||| GAAGGUCA--GCCC-------CUACAAAUGU	$A_4guggugA_2ccaauauA_3uA_1a$<br>$A_4gucaA_2A_3aA_1u$
CX43-miR-30e-5p	NNUGGUGUGGGCCAAUAUGGUGUUUACA<br>••|| •|| •|||||||| AGGUCAGUUCC--------UACAAAUGU	$nnA_2guA_3ccaauauA_4uA_1a$<br>$agA_2guA_3A_4aA_1u$
CX43-miR-199a*	NAUCAUUGAUGCUUGAAUGAUAGAAUUUUAGUACUGUA<br>|•|| || ||| ||• ||||||| UUGGUUAC-ACGU-----CUG----------AUGACAU	$nA_2uA_5aA_4uugaauA_3aguuuuagA_1a$<br>$uA_2uA_5A_4uA_3A_1u$
EGFR-miR-128a	GGAAGUUGC--AUUCCUUUGUCUUCAAACUGUGA<br>••||| •| ||| ||||||| UUUUCUCUGGCCAAG------------UGACACU	$A_2uuA_4aA_3cuuugucuucaaA_1a$<br>$A_2ucA_4gccA_3A_1u$

Supplementary figures

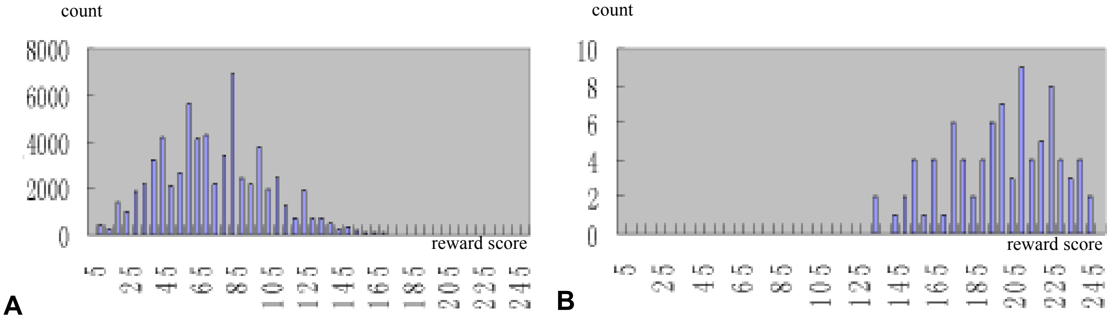

FIGURE 1. Histogram of the reward score values (*x*-axis) against the number of the corresponding targets (*y*-axis). (A) for targets that exclude the actual binding sites; (B) for the actual targets.

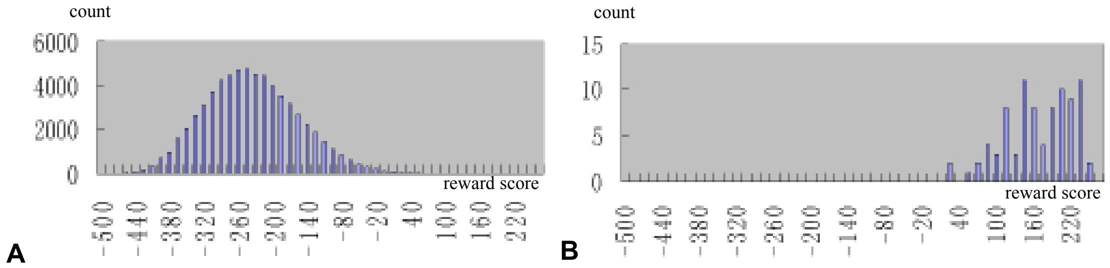

FIGURE 2. Histogram of the scoring function values (*x*-axis) against the number of corresponding targets (*y*-axis) (A) for targets that exclude the actual binding sites; (B) for the actual targets.

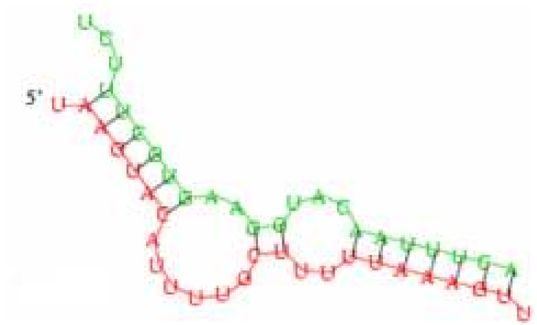

FIGURE 3. An example miR-mRNA duplex with bulges.

Input: miR(s) sequence and the 3'UTR sequence(s).
Output: the compacted sequence(s).
Step I. Check whether a given segment in 3'UTR provides complementary fit with the $T_2 \ldots T_7$ miR's segment (the complementary pairs do not include G:U).
If such segment exists then $T_2 \ldots T_7$ and the corresponding segment located in the 3'UTR sequence $m_N\, m_{N\text{-}1} \ldots m_2\, m_1$ are denoted by A_1 and go to step II; otherwise terminate.
Step II. For $i_1 \geq 9$, search for the longest segment $T_{i_1} \ldots T_{i_1+p_1}$ in the miR that satisfies the following two conditions:
1. $p_1 \geq 2$ and i_1 - 8 ≤ 15
2. there exists a segment $m_{N-j_1} \ldots m_{N-j_1-p_1}$ in the 3'UTR sequence $m_N\, m_{N\text{-}1} \ldots m_2\, m_1$ after A_1 satisfying: (1) $j_1 + p_1 -$ end_of_$A_1 \leq 15$; and (2) $m_{N-j_1} \ldots m_{N-j_1-p_1}$ is complementary with $T_{i_1} \ldots T_{i_1+p_1}$ (the complementary pairs may include G:U)

If $T_{i_1} \ldots T_{i_1+p_1}$ and $m_{N-j_1} \ldots m_{N-j_1-p_1}$ exist, then they are denoted by A_2 and go to step III; otherwise terminate.
Step III. Find A_3 by scanning for the longest segment $T_{i_2} \ldots T_{i_2+p_2}$ that satisfies the following two conditions:
1. $T_{i_2} \ldots T_{i_2+p_2}$ is complementary with segment $m_{N-j_2} \ldots m_{N-j_2-p_2}$ that is located in $m_N\, m_{N\text{-}1} \ldots m_2\, m_1$ and is sandwiched between A_1 and A_2
2. the largest value of $p_2 \geq 2$ is found

If $T_{i_2} \ldots T_{i_2+p_2}$ exists then we denote $T_{i_2} \ldots T_{i_2+p_2}$ and $m_{N-j_2} \ldots m_{N-j_2-p_2}$ by A_3 and go to step IV; otherwise go to step V.
Step IV. Search for A_4 (and A_5) using the following two sub-procedures:
Step IVa. Search for A_4 between A_1 and A_3, and if A_4 exists then go to step IVb to search A_5; otherwise go to step IVb to search A_4.
Step IVb. Search for A_4 (or A_5) between A_1 and A_4, and if A_4 (or A_5) exists then stop; otherwise go to **Step V**.
Step V. If the segment $A_1 \ldots A_2$ in the 3'UTR sequence satisfies L(A_1,A_2) < 47, where L(A_1,A_2) is the total number of nts within $A_1 \ldots A_2$, then go to **Step VI**; otherwise terminate.
Step VI. Search for the longest segment $T_{i_2} \ldots T_{i_2+p_2}$ between A_2 and the end of miR that satisfies the following two conditions:
1. $T_{i_2} \ldots T_{i_2+p_2}$ is complementary with a segment $m_{N-j_2} \ldots m_{N-j_2-p_2}$ after A_2 satisfying $j_2 + p_2 -$ end of $A_2 \leq 15$;
2. the largest value of $p_2 \geq 2$ is found

Denote $T_{i_2} \ldots T_{i_2+p_2}$ and $m_{N-j_2} \ldots m_{N-j_2-p_2}$ by A_3 and go to step VIa; otherwise go to step VII.
Step VIa. Search for A_4 between A_2 and A_3, and if A_4 exists then go to step VIb; otherwise terminate.
Step IVb. Search for A_5 between A_2 and A_4 or between A_4 and A_3; terminate after this search.
Step VII. If A_j exists in the region of 3'UTR sequence after A_2 such that L(A_1,A_j) < 47 then keep the A_j, and search for A_{i+1} between A_2 and A_i; otherwise terminate.

FIGURE 4. Pseudo-code of the 2D-coding algorithm.
